# Intestinal motility shapes spatiotemporal patterns of enterohemorrhagic *Escherichia coli* colonization and virulence gene expression in the host gut

**DOI:** 10.1128/iai.00657-25

**Published:** 2026-04-27

**Authors:** S. Dutta, W. Galdamez, A. T. Nguyen, M. A. Odem, L. Thompson, R. E. Bosserman, A. M. Krachler

**Affiliations:** 1 Department of Microbiology and Molecular Genetics, McGovern Medical School, The University of Texas Health Science Center at Houston 12340, Houston, Texas, USA; 2 M.D. Anderson Cancer Center UTHealth Graduate School of Biomedical Sciences, Houston, Texas, USA; University of Illinois Chicago, Chicago, Illinois, USA

**Keywords:** gut motility, virulence regulation, intestinal colonization, zebrafish, EHEC

## Abstract

Enterohemorrhagic *Escherichia coli* (EHEC) regulates the expression of virulence gene in response to diverse host environmental cues. The LEE pathogenicity island encodes a type III secretion system required for colonization and is responsive to chemical and mechanical stimuli. Prior *in vitro* studies have shown that the LEE regulator GrlA responds to mechanical force and is required for mechanoregulation and colonization. The physiological role of gut motility as a cue for LEE regulation in a living host, however, has not been directly studied. Using larval zebrafish, a genetically tractable and optically transparent model with conserved gut architecture and functional peristalsis, we examined EHEC colonization and LEE expression in hosts with normal (wild-type) or disrupted (*sox10* mutant or atropine-treated) gut motility. In wild-type larvae, bacterial colonization and LEE expression followed distinct spatial and temporal patterns, whereas in dysmotile hosts, these patterns were disrupted. Loss of spatial and temporal restriction of LEE expression and colonization was associated with reduced bacterial fitness and increased host survival. These findings indicate that host gut motility contributes to the spatiotemporal organization of virulence activation and colonization *in vivo* and that these patterns are important for pathogenesis.

## INTRODUCTION

Enterohemorrhagic *Escherichia coli* (EHEC), a subset of Shiga toxin-producing *E. coli* (STEC), is a foodborne pathogen capable of causing severe gastrointestinal illness, including hemorrhagic colitis and hemolytic uremic syndrome (1). A hallmark of EHEC is its ability to tightly regulate virulence gene expression in response to environmental cues encountered during gut colonization (2–4). Central to this process is the locus of enterocyte effacement (LEE), a horizontally acquired pathogenicity island that encodes a type III secretion system (T3SS), effector proteins, and associated transcriptional regulators (5, 6). The LEE-encoded T3SS mediates intimate adherence of EHEC to intestinal epithelial cells, promoting the formation of attaching and effacing (A/E) lesions that facilitate colonization and disease progression (7, 8).

Expression of the LEE is tightly controlled by both host and bacterial factors, ensuring that energy-intensive virulence programs are only activated under appropriate conditions (9). Environmental cues such as oxygen tension, bile salts, temperature, short-chain fatty acids, and carbon source availability modulate LEE activity through global regulators including Ler (encoded by LEE1), GrlA/GrlR (LEE7), and the quorum sensing regulator QseA (10–13). In addition to these well-studied chemical cues, recent studies have highlighted the role of physical forces in shaping bacterial behavior, with shear stress and surface contact emerging as important regulators of LEE expression (4, 14). More recent experiments have shown that the LEE activators AirA and GrlA function as mechanosensitive elements, integrating physical stimuli into the LEE regulatory network (14, 15). However, whether such mechanical cues are sensed and acted upon within the dynamic, peristaltic environment of a living intestine—and how they intersect with other regulatory inputs—remains poorly understood.

Animal models are essential for elucidating the *in vivo* dynamics of host-pathogen interactions and the complex environmental signals encountered by enteric pathogens. Zebrafish (*Danio rerio*) larvae have emerged as a powerful model for studying intestinal colonization and host responses to microbial infection (16). The optical transparency of larval zebrafish allows for non-invasive, high-resolution imaging of both host tissues and fluorescently labeled microbes in real time (17). Zebrafish possess a functional innate immune system and a segmented gastrointestinal tract with peristaltic motility, mucosal barriers, and conserved epithelial structures (18–20). These features, combined with their genetic tractability, make zebrafish uniquely suited for dissecting how biomechanical forces, such as peristalsis, shape bacterial physiology during infection and how altered motility states impact host–microbe interactions (21–23).

Zebrafish larvae have been adapted as a model for food-borne EHEC infection that recapitulates key aspects of pathogenesis (24, 25). EHEC colonizes discrete intestinal niches in the larval zebrafish gut, induces expression of the LEE-encoded T3SS, and causes dose-dependent host morbidity and mortality (15, 24). Thus, zebrafish provide a physiologically relevant vertebrate intestinal environment in which EHEC virulence regulation, colonization dynamics, and transmission can be studied *in vivo* with high spatial and temporal resolution.

Recent work has established that host-driven physical forces are potent determinants of gut microbial organization: Schlomann et al. (26) demonstrated that gut motility in zebrafish larvae generates spatial and temporal heterogeneity in microbial communities (26), while Wiles et al. (27) showed that the enteric nervous system can modulate bacterial population dynamics through motility-dependent spatial structuring (27). Complementary studies, including those by Stephens et al. (28), have revealed that mechanical forces, such as peristalsis, influence bacterial positioning and persistence in the gut, reinforcing the view that motility is a central organizer of microbial spatial patterning (28).

While these studies have largely centered on commensal communities, the same mechanisms are likely to influence pathogenic bacteria. We therefore sought to extend this framework to examine whether motility-driven spatial organization in the gut also shapes EHEC virulence gene expression and colonization. EHEC colonizes the larval zebrafish gut and induces expression of virulence genes, including the LEE, during gut colonization (8, 24); yet, the host-derived signals governing this activation remain incompletely defined. We hypothesized that gut motility itself may act as a physiological signal coordinating spatial and temporal aspects of EHEC virulence activation, potentially through both direct mechanosensing and indirect effects of gut motility on chemical gradients in the intestinal environment.

We used time-lapse fluorescence imaging of EHEC expressing transcriptional reporters for the LEE1-encoded virulence master regulator Ler (*ler:gfp*) and a constitutive colonization marker (mCherry) to examine how gut motility influences virulence regulation and bacterial colonization during infection. We compared wild-type (AB) zebrafish larvae with *sox10* mutants, which lack a functional enteric nervous system and exhibit markedly reduced gut motility (29, 30). These mutants provide a genetic model of gut dysmotility, allowing us to test whether physical forces generated by intestinal peristalsis influence bacterial behavior *in vivo*. We also pharmacologically impaired motility in wild-type larvae using the antispasmodic drug atropine (31) to determine whether motility-associated effects on EHEC colonization and gene expression are recapitulated by chemical disruption.

We found that in wild-type zebrafish, EHEC colonization and LEE gene expression were both spatially and temporally patterned. In contrast, *sox10* mutants exhibited disorganized colonization and dysregulated LEE expression, accompanied by reduced bacterial fitness and virulence. These phenotypes were mimicked in atropine-treated larvae, providing convergent evidence that gut motility is a physiological cue affecting EHEC colonization and pathogenesis in the host intestine.

## RESULTS

### Spatiotemporal patterning of EHEC virulence gene expression and colonization in wild-type fish

To determine whether gut motility shapes the spatial and temporal regulation of EHEC virulence gene expression during colonization, we imaged wild-type AB zebrafish larvae infected at 6 days post-fertilization (dpf) with a dual-reporter strain expressing GFP driven by the promoter of the LEE1-encoded virulence master regulator Ler (*ler:gfp*) and constitutive mCherry to label all bacteria.

**Fig 1:**
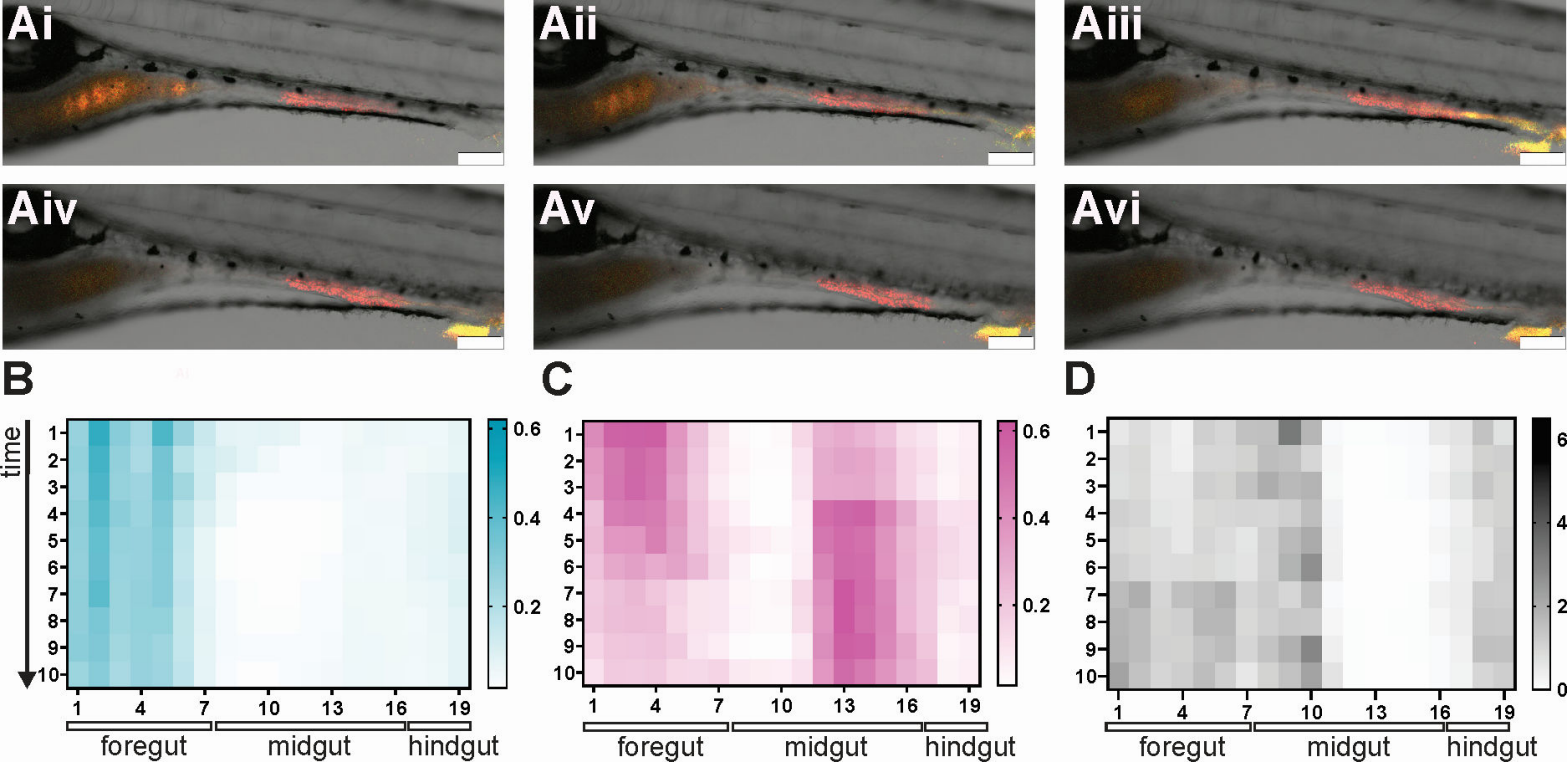
Gut motility supports spatially restricted activation of EHEC virulence genes in wild-type zebrafish larvae. (**A**) Selected still images at *t* = 1, 2, 4, 6, 8, and 10 h (I–VI) from time-lapse imaging of wild-type AB larvae infected with dual-reporter EHEC (*ler::gfp*, mCherry) reveal a midgut colonization followed by posterior expansion and expulsion. GFP fluorescence, indicating LEE activation, is localized to discrete hotspots along the gut axis (foregut and expelled bacteria). Scale bar: 100 µm. (**B**) Quantitative intensity heatmap of green fluorescence (*ler:gfp*) intensity along the gut axis (region of interest 1–19, left to right) and over time (3–13 hpi, top to bottom). ROIs 1–7 correspond to foregut, ROIs 8–16 to midgut, and ROIs 17–19 to hindgut. ROI 20 was defined within the field of view, but outside the imaged larvae, and was used as background normalization. (**C**) Quantitative heatmap of red fluorescence intensity (EHEC:mcherry colonization) along gut axis and over time. (**D**) Quantitative heatmap of GFP/mcherry intensity ratio as a control. *N* = 9 fish (three independent experiments).

Time-lapse imaging revealed a dynamic pattern of colonization and virulence activation: EHEC initially localized to the foregut, from which it was gradually cleared several hours after initiating infection. Bacteria rapidly colonized a discrete segment at the midgut-hindgut boundary (ROIs 12–17), where they established a stable population, and non-adherent EHEC were expelled in bursts and localized outside the intestine as fecal matter (Fig. 1A; Fig. S1D; Movie S1). Strikingly, GFP fluorescence—indicating LEE activation— was not uniformly distributed throughout the gut either, but instead localized to discrete zones in the foregut and rectal region/fecal matter, while the colonized midgut zone showed a low level of GFP expression.

Quantitative analysis of fluorescence intensity distribution along the anterior–posterior gut axis confirmed distinct peaks of GFP intensity at defined locations, consistent with spatially restricted virulence gene expression (Fig. 1B). LEE expression was highest at earlier time points and gradually decreased over time (Fig. 1B). Heatmap analysis of mCherry fluorescence intensity across several fish showed that bacterial density in the foregut decreased over time, while colonization of the posterior midgut initially increased and then remained stable over time (Fig. 1C). Analysis of the GFP/mCherry intensity ratio demonstrated that the *ler:gfp* expression pattern was not driven by bacterial density alone, since patterning persisted in this map (Fig. 1D). This was further confirmed by the absence of correlation between GFP and mCherry mean intensities across ROIs (Fig. S1A). In addition to spatial patterning, there was a pronounced temporal component in *ler:gfp* and mCherry fluctuations. When GFP/mCherry intensity ratios were averaged across all ROIs to assess time dependence, the overall signal displayed clear fluctuations over time, indicating dynamic modulation of virulence gene expression and colonization rather than a steady-state pattern (Fig. S1C). Together, these findings establish that in wild-type larvae with normal peristalsis, EHEC virulence gene regulation and bacterial colonization follow a distinct spatiotemporal pattern.

### EHEC virulence gene expression and colonization are dysregulated in sox10 fish with impaired gut motility

The zebrafish s*ox10^−/^*^−^ line lacks an enteric nervous system due to loss of neural crest–derived enteric neurons, making it a well-established model for studying the role of gut innervation in host–microbe interactions (23, 32). These larvae exhibit severe defects in gut motility and prolonged transit times, making them an ideal system to probe how coordinated peristalsis shapes EHEC spatial colonization and virulence regulation. Consistent with prior work showing motility defects in this line (32), functional transit assays using food spiked with a fluorescent tracer (33) confirmed significantly prolonged gut transit times compared to wild-type larvae (Fig. S2A).

**Fig 2:**
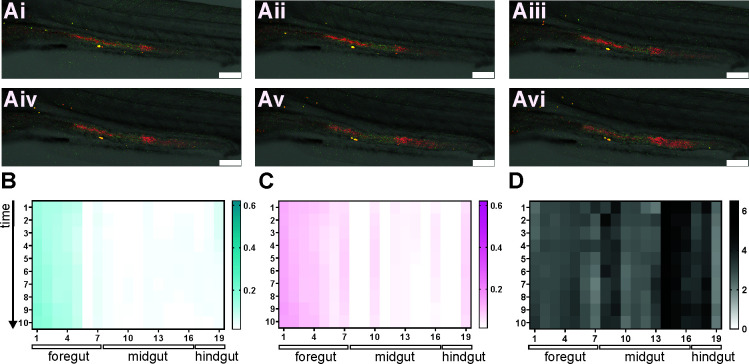
Loss of coordinated gut motility in *sox10* mutant zebrafish disrupts spatial patterning of EHEC colonization and virulence activation. (**A**) Selected still images at *t* = 1, 2, 4, 6, 8, and 10 h (I–VI) from time-lapse imaging of *sox10* larvae infected with dual-reporter EHEC (*ler:gfp*, mCherry) reveal a diffuse pattern of colonization and virulence gene expression across time. GFP fluorescence, indicating LEE activation, is localized to discrete hotspots along the gut axis (foregut and expelled bacteria). Scale bar: 100 µm. (**B**) Quantitative intensity heatmap of green fluorescence (*ler:gfp*) intensity along gut axis (region of interest 1–19, left to right) and over time (1–10 hpi, top to bottom). ROIs 1–7 correspond to foregut, ROIs 8–16 to midgut, and ROIs 17–19 to hindgut. ROI 20 was defined within the field of view, but outside the imaged larvae, and was used as background normalization. (**C**) Quantitative heatmap of red fluorescence intensity (EHEC:mcherry colonization) along gut axis and over time. (**D**) Quantitative heatmap of GFP/mCherry intensity ratio as a control. *N* = 9 fish (three independent experiments).

Both wild-type and *sox10* larvae initially took up similar amounts of EHEC during the early stages of infection (Fig. S3). Despite this, *sox10* larvae infected with *ler:gfp*/mCherry EHEC showed significantly lower colonization levels and LEE expression at 3–13 hpi (Fig. 2A; Movie S2). Quantification of anterior–posterior GFP and mCherry distribution over time confirmed the absence of a clear spatiotemporal pattern and a marked reduction in overall fluorescence intensity (Fig. 2B and C). Unlike in wild-type fish (Fig. 1), analysis of the GFP/mCherry intensity ratio in *sox10* fish (Fig. 2D) showed no discernible spatial pattern, and GFP and mCherry intensities were strongly correlated, suggesting that residual LEE expression is largely density-driven (Fig. S1B). The temporal fluctuations in GFP/mCherry intensity observed in wild-type larvae were markedly reduced or absent in *sox10* fish, indicating a loss of the dynamic expression pattern (Fig. S1C). Collectively, these findings indicate that in the absence of coordinated gut motility, EHEC fails to establish discrete colonization niches, and virulence gene expression is both reduced and temporally flattened. While the reduced LEE expression may reflect a direct mechanosensory response to impaired peristalsis, we cannot exclude indirect effects mediated by altered luminal conditions in *sox10* larvae. Nevertheless, the clear contrast with wild-type patterns provides strong evidence that gut motility is a critical determinant of EHEC spatial and temporal virulence regulation.

### Dysregulation of motility impairs EHEC fitness *in vivo*

**Fig 3:**
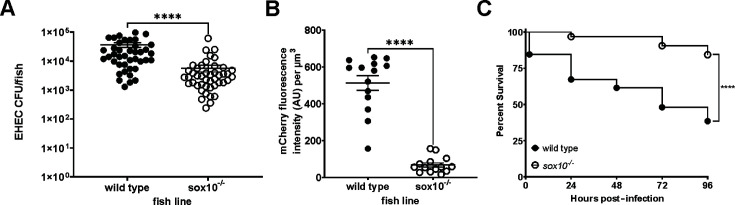
Disrupted gut motility in *sox10* fish reduces EHEC colonization and attenuates infection. (**A**) EHEC CFUs recovered at 24 h post-infection from individual fish (*n* = 45 fish/group over three independent experiments), shown as means ± SEM. (**B**) Total mCherry fluorescence intensity per µm^3^, as a proxy for bacterial colonization, was determined from z-stacks of infected fish at 24 hpi (*n* = 14 fish/group over three independent experiments), shown as means ± SEM. (**C**) Survival of wild-type and *sox10* larvae infected with a high dose of EHEC (*n* = 150 fish/group over three independent experiments) was analyzed using a Kaplan-Meier plot. *Sox10* results were compared to wild-type using unpaired *t*-test for panels A and B, and a log-rank (Mantel-Cox) test for panel C. ****P ≤ 0.0001.

To test whether the disrupted colonization and virulence gene expression patterns observed in *sox10* larvae affect EHEC fitness and infection outcome, we quantified EHEC burden and host survival in both wild-type and *sox10* larvae. Despite both lines taking up similar initial inocula immediately following infection (Fig. S3), *sox10* larvae harbored significantly fewer colony-forming units (CFUs) of EHEC at 24 h post-infection relative to their wild-type siblings (Fig. 3A). This reduced burden in *sox10* compared to wild-type ABs was further corroborated by quantifying the total mCherry fluorescence intensity across the gut 24 h post-infection (Fig. 3B), as well as by the reduced intensity observed throughout the time-lapse imaging (Fig. 2C). This reduction in bacterial burden correlated with improved host survival. While standard-dose infections rarely cause mortality, at higher bacterial burdens, more than 60% of wild-type larvae succumbed to infection by 96 h post-infection, whereas *sox10* larvae exhibited significantly enhanced survival under the same conditions (Fig. 3C). There was no difference in survival between uninfected wild-type and *sox10* larvae (Fig. S4A), indicating that the enhanced survival of the mutant is infection-dependent rather than due to baseline physiological differences. These results indicate that intact gut motility creates a microenvironment that promotes EHEC colonization, virulence gene activation, and pathogen fitness, ultimately contributing to host mortality. Disruption of motility, as seen in *sox10* larvae, impairs these processes, reducing pathogen fitness and improving host outcomes.

Because *ler* encodes the master regulator of the LEE pathogenicity island, we next asked whether host killing in this model is dependent on an intact T3SS. Previous work using the zebrafish infection model demonstrated that repression of the EHEC T3SS significantly attenuates larval killing (15, 24), supporting a central role for T3SS activity in pathogenesis. To directly address this question in the context of the current strain background and infection conditions, we performed comparative killing assays using wild-type EHEC and a T3SS-deficient Δ*ler* mutant. Consistent with both prior work and the virulence expression patterns observed here, loss of *ler* resulted in a marked attenuation of host killing (Fig. S5), indicating that EHEC-mediated mortality in the zebrafish intestine is dependent on a functional T3SS. Together, these findings demonstrate that gut motility promotes EHEC virulence not only by shaping colonization dynamics but also by enabling Ler-dependent activation of the T3SS that is required for pathogenic outcomes *in vivo*.

### Pharmacological inhibition of gut motility phenocopies the sox10 mutant

*Sox10* fish display multiple developmental and physiological abnormalities beyond the loss of enteric neurons, including craniofacial defects and disrupted intestinal homeostasis (29, 32, 34). Their aperistaltic gut is characterized by microbial dysbiosis, altered luminal pH, and mild baseline inflammation (23, 32), making it difficult to distinguish direct effects of motility from secondary consequences of broader host dysfunction.

To independently test whether gut motility directly influences EHEC colonization dynamics and virulence gene expression, we employed pharmacological inhibition of intestinal peristalsis using atropine, a well-characterized muscarinic acetylcholine receptor antagonist (35). Atropine blocks parasympathetic signaling, leading to decreased smooth muscle contractility and reduced gut motility—effects that have been extensively documented in various vertebrate models, including zebrafish (31, 33). Prior studies have established zebrafish larvae as a valuable model for studying drug-induced modulation of gut motility, demonstrating that compounds, such as atropine, reliably impair peristalsis without overt toxicity (31, 36). Consistent with these reports, treatment of wild-type larvae with 4.2 µM atropine caused a significant decrease in peristalsis and transit (Fig. S2B), effectively phenocopying the gut motility defects observed in *sox10* mutants. Treatment with 0.42 µM atropine had no significant effect on motility (Fig. S2B). Thus, 4.2 µM was used as treatment concentration in subsequent experiments.

**Fig 4:**
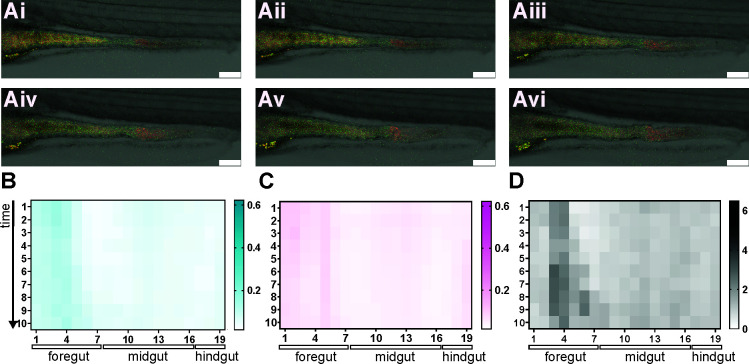
Pharmacological inhibition of motility phenocopies EHEC infection patterns of *sox10* mutants. (**A**) Representative still images at *t* = 1, 2, 4, 6, 8, and 10 h (I–VI) from time-lapse imaging of wild-type larvae treated with 4.2 µM atropine and infected with dual-reporter EHEC (*ler:gfp*, mCherry) show attenuated virulence gene expression and a diffuse colonization pattern. Scale bar: 100 µm. (**B**) Quantitative intensity heatmap of green fluorescence (*ler:gfp*) intensity along gut axis (region of interest 1–19, left to right) and over time (1–10 hpi, top to bottom). ROIs 1–7 correspond to foregut, ROIs 8–16 to midgut, and ROIs 17–19 to hindgut. ROI 20 was defined within the field of view, but outside the imaged larvae, and was used as background normalization. (**C**) Quantitative heatmap of red fluorescence intensity (EHEC:mcherry colonization) along gut axis and over time. (**D**) Quantitative heatmap of GFP/mCherry intensity ratio as a control. *N* = 9 fish (three independent experiments).

When infected with the *ler:gfp*/mCherry EHEC reporter strain, atropine-treated larvae exhibited a substantially reduced, spatially unpatterned LEE expression and decreased colonization with a dispersed localization (Fig. 4A; Movie S3), closely mirroring the dysregulation and disruption of spatiotemporal patterning seen in the *sox10* larvae (Fig. 2). Quantitative analysis confirmed significant reductions and disruption of patterning in both GFP fluorescence intensity (Fig. 4B) and mCherry signal (Fig. 4C) relative to untreated controls. Ratiometric analysis of fluorescence intensity distribution (Fig. 4D) and correlation analysis of GFP and mCherry signals (Fig. S7A) also showed a strong interdependence of GFP and mCherry intensities, suggesting that GFP signal is driven by bacterial density alone. Unlike in untreated wild-type fish, there was no significant temporal variation in fluorescence intensity in atropine-treated AB larvae (Fig. S7B)

**Fig 5:**
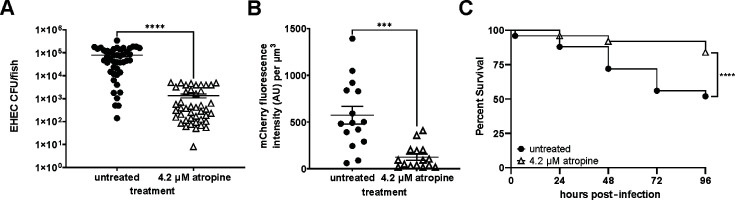
Disruption of gut motility with atropine reduces EHEC fitness and attenuates infection outcomes. (**A**) EHEC CFUs recovered at 24 h post-infection from individual AB larvae either left untreated or treated with 4.2 µM atropine (*n* = 45 fish/group over three independent experiments), shown as means ± SEM. (**B**) Total mCherry fluorescence intensity per µm^3^, as a proxy for bacterial colonization, was determined from z-stacks of infected fish at 24 hpi (*n* = 15 fish/group over three independent experiments), shown as means ± SEM. (**C**) Survival of untreated and atropine-treated wild-type larvae infected with a high dose of EHEC (*n* = 75 fish/group over three independent experiments) was analyzed using a Kaplan-Meier plot. Results for untreated and atropine-treated fish were compared using unpaired *t*-test for panels A and B, and a log-rank (Mantel-Cox) test for panel C. ****P ≤ 0.0001.

Consequently, atropine treatment attenuated bacterial fitness and host mortality: atropine-treated fish harbored fewer EHEC CFUs (Fig. 5A) and a lower level of total mCherry EHEC at 24 h post-infection (Fig. 5B). This reduced bacterial fitness led to enhanced host survival post-infection when fish were subjected to a higher EHEC dose and assessed for mortality (Fig. 5C). Atropine treatment alone did not affect larval survival in the absence of infection (Fig. S4B) or EHEC growth (Fig. S6), indicating that altered infection outcomes are attributable to disrupted gut motility rather than drug toxicity. Together, these findings provide convergent pharmacological evidence that gut motility serves as a critical factor structuring the host intestinal niche and regulating pathogen virulence gene expression and colonization *in vivo*.

## DISCUSSION

Our study highlights gut motility as a critical physiological cue influencing the regulation of EHEC virulence gene expression during intestinal colonization. By combining genetic and pharmacological models of impaired motility in zebrafish, we provide evidence that peristalsis helps spatially organize bacterial colonization and modulates expression of key virulence determinants through the virulence master regulator Ler. Importantly, the Ler-dependent virulence patterns we observe are functionally relevant for pathogenesis, as loss of *ler* in the current strain background strongly attenuates host killing in the zebrafish model. This is consistent with our prior work demonstrating that repression of the EHEC T3SS significantly reduces larval mortality (15, 24), reinforcing that LEE/T3SS activity is a critical determinant of disease outcome in the zebrafish model.

This expands the current understanding of host environmental signals beyond classical chemical cues, positioning gut motility as a fundamental regulator within the host-pathogen dialog.

Our findings align with prior *in vitro* work demonstrating shear stress as an inducer of LEE expression, likely mediated by mechanosensitive regulators such as AirA and GrlA (4, 14, 15). Similar induction of virulence genes by mechanical force or flow sensing has also been observed in pathogens such as *Pseudomonas aeruginosa* (37, 38). While these studies show that physical forces can be sensed by bacterial regulatory networks, the relevance of such pathways during host colonization was previously unclear. Here, we provide a physiological context in which gut peristalsis generates mechanical stimuli that spatially and temporally pattern virulence gene activation, emphasizing that EHEC integrates both chemical and biomechanical signals to finely tune its pathogenic program.

The zebrafish model has proven instrumental for dissecting the dynamics of host–microbe interactions in a living vertebrate host. Prior research has demonstrated the importance of gut motility in shaping microbial community structure and function, including modulation of bacterial colonization niches and impacts on host immune responses (23, 32, 39). Additional work further suggested that peristalsis reduces bacterial aggregation and helps distribute microbes along the gut axis (40). Prior characterization of bacterial growth in the zebrafish gut showed that a commensal species exhibited logistic growth *in vivo* (41). In contrast, our observations for EHEC reveal that this pathogen colonizes discrete niches early during infection and subsequently maintains a relatively stable bacterial load, highlighting distinct growth dynamics for a virulent organism. However, in line with this study, our results show spatial heterogeneity for bacterial colonization. Our results further extend the existing body of work by demonstrating that gut motility not only influences bacterial spatial distribution but also directly impacts pathogen gene regulation and fitness.

While our use of *sox10* mutants and atropine treatment effectively models gut dysmotility, there are some caveats to consider. The *sox10* mutation disrupts enteric nervous system development, potentially affecting host immune and epithelial functions beyond motility, which might indirectly influence bacterial colonization or virulence (32). Similarly, atropine has systemic effects on cholinergic signaling that could modulate host physiology beyond peristalsis (42, 43), complicating interpretation. Thus, it remains difficult to fully disentangle direct mechanosensory effects from indirect effects of peristalsis on intestinal parameters such as pH and other gradients. Bacterial mutants compromised in their ability to sense mechanical cues exist (15) but poorly colonize the gut at baseline, further limiting the ability to fully distinguish between direct mechanical responses to peristalsis and indirect effects. However, the convergence of results from genetic and pharmacological manipulation of gut motility strongly suggests that peristalsis may directly impact EHEC gene regulation and colonization patterns. In addition, bacterial flagellar motility may contribute to intestinal position and colonization dynamics, as flagellar gene expression in EHEC is inversely regulated with the LEE and is typically repressed upon induction of the T3SS (44, 45). Thus, alterations in gut motility could also influence bacterial spatial distribution by modulating the balance between motile and adherent states.

Additionally, although our imaging-based analyses reveal spatial patterning of LEE expression, the precise molecular mechanisms by which mechanical forces are transduced by EHEC *in vivo* remain to be elucidated. Candidate regulators, such as AirA and GrlA, may act as mechanoresponsive transcriptional activators, but direct observation of their specific role in the host environment is still lacking. Recent work has shown that both inactivation and constitutive activation of GrlA are detrimental to bacterial fitness and colonization of the host gut (15). Further investigation using bacterial mutants deficient in mechanosensitive pathways will clarify details of this pathway *in vivo*, although, as mentioned above, these studies are complicated due to low baseline colonization in the absence of mechanoregulators (15).

From a translational perspective, our findings highlight the importance of biomechanical cues in regulating pathogen virulence. However, the complexity of host motility’s role in maintaining intestinal homeostasis and microbiota balance warrants careful consideration, as broadly impairing peristalsis might have unintended consequences. Clinical studies have demonstrated that administration of antimotility agents, such as loperamide (Imodium), increases the risk of developing hemolytic uremic syndrome (46). As such, modulation of gut motility in the present study is used strictly as a mechanistic tool to interrogate host-pathogen interactions, rather than as a therapeutic strategy. Instead, bacterial mechanoresponsive pathways may present a viable target for development of anti-infectives, and recent screens have identified small-molecule GrlA inhibitors (47).

In conclusion, this work underscores gut motility as a spatial and temporal organizer of enteric infection microenvironments and a potent physiological signal integrated by pathogens to coordinate virulence gene expression. The integration of biomechanical cues with traditional chemical signals likely represents a generalizable strategy employed by intestinal microbes, opening new avenues for understanding and ultimately intervening in host–microbe interactions.

## MATERIALS AND METHODS

### Zebrafish lines and husbandry

All zebrafish experiments were conducted in accordance with the Guide for the Care and Use of Laboratory Animals and were approved by the Institutional Animal Welfare Committee of the University of Texas Health Science Center, Houston (protocol number AWC-22-0088). The zebrafish (*Danio rerio*) used in this study were wild-type AB and *sox10***^−/^**^−^ larvae, which lack a functional enteric nervous system and exhibit impaired gut motility (29). The *sox10* fish were maintained as a heterozygous breeding colony (*sox10^−/+^*), and following breeding, experimental larvae (*sox10^−/^*^−^) were identified by a lack of pigmentation and separated from the remainder of the clutch. Adult zebrafish were housed in a recirculating aquaculture system at the University of Texas Health Science Center at Houston Laboratory Animal Medicine and Care facility under standard conditions (28°C, pH 7.5, 14:10 h light:dark cycle). Fish were maintained at a density of up to five adult fish per liter of system water and fed GEMMA Micro (Skretting) twice daily, and embryos were obtained by natural spawning. Fertilized embryos were briefly surface-sterilized in 0.05% sodium hypochlorite (Sigma-Aldrich) for 30 s, rinsed three times in sterile embryo (E3) medium, and raised in petri dishes containing E3 (10 mM HEPES, 5 mM NaCl, 0.17 mM KCl, 0.4 mM CaCl₂, 0.67 mM MgSO₄, pH 7.4) at 28°C on a 14:10 h light:dark cycle. PTU was added to embryos used for time-lapse imaging to inhibit pigmentation. For experiments, zebrafish larvae were used at 6–7 days post-fertilization (dpf), corresponding to the developmental window when the intestine is fully formed and peristaltic motility is active (18).

### Bacterial strains and growth conditions

The bacterial strain background used in this study is EHEC strain 86-24 (48). The Δ*ler* deletion strain was constructed using gene doctoring, as previously described (49). For monitoring colonization and gene expression *in vivo*, strains were transformed with plasmid pDP151-mCherry (AmpR). To visualize induction of the LEE1-encoded regulator Ler, strains were transformed with plasmid *ler:gfp* (TetR) (4). Strains were grown in LB broth supplemented with 100 µg/mL ampicillin or 34 µg/mL tetracycline, where appropriate, at 37°C with gentle shaking.

### Zebrafish infections

Paramecia were propagated as described previously 1 day prior to infection experiments and maintained by passage every 2 weeks to sustain live cultures. Loading of paramecia with *E. coli* was performed as described (25). On the day of infection, paramecia were co-cultured with bacteria for 2 h and thoroughly washed to remove extracellular EHEC, as described (50). *E. coli*–loaded paramecia were counted using an automated cell counter (Life Technologies Countess II) and adjusted to a final concentration of 2 × 10^5^ paramecia/mL in E3 medium. For survival experiments, the larvae were exposed to a higher dose of EHEC by adjusting the paramecia concentration to 2 × 10^6^ paramecia/mL. Zebrafish larvae (6 dpf) were exposed to loaded paramecia for 2 h at 30°C in 6-well plates. After 2 h, larvae were anesthetized in E3 medium containing 0.16 mg/mL tricaine, thoroughly washed in fresh E3 to remove excess paramecia and external bacteria, and transferred to fresh plates containing E3.

### Bacterial burden quantification

Initial bacterial burden in zebrafish larvae was assessed immediately after co-incubation of larvae with paramecia. Bacterial colonization was assessed at 24 h post-infection. To quantify EHEC burden, infected larvae were euthanized in 1.6 mg/mL tricaine, washed thoroughly in sterile E3, and incubated with 100 μL of 1 mg/mL filter-sterilized pronase solution at 37°C for 60 min with intermittent vortexing to aid tissue breakdown. Larval tissues were homogenized by repeated passage through a 31-gauge needle attached to a 1 mL syringe. Homogenates were serially diluted in sterile PBS and plated on EHEC CHROMagar O157 plates. EHEC colonies are readily distinguished from other gut-associated bacteria by their mauve color. Plates were incubated at 30°C for 24 h and then held at room temperature for an additional 24 h to permit full colony development. EHEC burden was expressed as CFU per larva. All data were analyzed in GraphPad Prism (version 9), and statistical significance was determined using tests indicated in the figure legends.

### Zebrafish survival analysis

Survival was assessed following treatment of larvae with 0––4.2 µM atropine alone, or following atropine treatment followed by infection as described above, using a high dose of EHEC-loaded paramecia (2 × 10^6^ paramecia/mL). Survival was assessed at 2 and 24 h post-infection and every 24 h thereafter up to 96 h post-treatment by observing the presence or absence of operculum movement using an Olympus SZX10 stereomicroscope. Dead larvae were removed, and survivors were transferred into a fresh dish. The analysis was done on groups of at least 15 fish each and repeated three times. Data were analyzed using the Kaplan-Meier method, and statistical significance was assessed using the Mantel-Cox test.

### Confocal microscopy and analysis of fluorescence

An Olympus Fluoview FV3000 confocal laser scanning microscope was used for live imaging. Following infection as described above, the larvae were anesthetized with 0.16 mg/mL tricaine, washed thoroughly, and embedded with 1% low-melting-point agarose in E3 media containing 0.16 mg/mL tricaine in a 6-well glass-bottom plate. The mounted larvae were imaged using 10× or 20× objectives and excitation wavelengths of 488 nm and 561 nm, with 1 μm step-size image stacks. Images were recorded every hour for up to 10 h, corresponding to approximately 3–13 hpi due to the time required to wash, embed, and set up larvae for time-lapse imaging. To quantify bacterial fluorescence in the intestine, seven dpf AB wild-type and *sox10^−^/*^−^ larvae were infected as previously described and imaged using the confocal microscope for 10 h. Images were deconvolved in CellSens using the constrained iterative algorithm and three iterations. Nineteen ROIs, each measuring 100 μm high by 55 μm wide, were placed along the intestine, with ROIs 1–7 covering the foregut, ROIs 8–16 for the midgut, and ROIs 17–19 for the hindgut (Fig. S1D). A separate 100 μm by 55 μm ROI (ROI 20) was placed outside of the larvae to correct for background fluorescence. Fluorescence intensity values for the mCherry and GFP channels were extracted for each ROI at each time point, adjusted for the total number of z-slices to account for imaged volume, and had background fluorescence subtracted. The resulting intensities were scaled between 0 and 1 across all data sets to allow direct comparisons. Fluorescence channels were normalized independently. Mean heat maps using these adjusted and normalized values were generated with Prism 9.5.1.

### Preparation of fluorescent food and transit assay

Fluorescent tracer food was prepared as previously described (33) with minor modifications. The ratio of food to tracer was maintained according to the published protocol, but Gemma Micro 75 ZF (Skretting) was used as the base food and supplemented with fluorescent red, amine-modified polystyrene beads (1 µm mean particle size, Sigma L2778). From 5 dpf, larvae were fed unlabeled Gemma twice daily. At 6 dpf, fish were fed 2 mg of tracer food for 2 h. Subsequently, fish with a tracer-filled bulb were sorted into batches of *n* = 5 per group (three independent experiments, total *n* = 15 fish/group). These fish were maintained in either standard E3 medium or E3 containing 0.42 or 4.2 µM atropine as indicated. Fish were imaged and transit scored at 2, 4, 6, and 24 h post-exposure. Transit zones were scored visually using the scheme described in reference (51), and data were analyzed by unpaired *t*-tests, comparing mean transit zones between the two groups at each time point.

## SUPPLEMENTAL MATERIAL

Supplemental material iai.00657-25-s0001.docx Fig. S1 to S7.

Movie S1 iai.00657-25-s0002.avi Infected AB larvae.

Movie S2 iai.00657-25-s0003.avi *sox10* larvae.

Movie S3 iai.00657-25-s0004.avi Atropine treated larvae.

ASM does not own the copyrights to Supplemental Material that may be linked to, or accessed through, an article. The authors have granted ASM a non-exclusive, world-wide license to publish the Supplemental Material files. Please contact the corresponding author directly for reuse.
