# Supplementary material for: Intestinal motility shapes spatiotemporal patterns of enterohemorrhagic Escherichia coli colonization and virulence gene expression in the host gut
Source: Infect Immun. 2026 Apr 27;94(6):e00657-25. doi: 10.1128/iai.00657-25 (PMC13248669; doi:10.1128/iai.00657-25)
Supplement: Supplemental material — Fig. S1 to S7. [file iai.00657-25-s0001.docx]

Supplemental Materials for

**Intestinal motility shapes spatiotemporal patterns of enterohemorrhagic Escherichia coli colonization and virulence gene expression in the host gut**

**Dutta S^1^, Galdamez W^1,2^, Nguyen AT****^1,2^, Odem MA^1,3^, Thompson L^1,2^, Bosserman RE^1^, Krachler AM^1,2,*^**

**^1^** Department of Microbiology and Molecular Genetics, McGovern Medical School, The University of Texas Health Science Center at Houston, Houston, Texas.

**^2^** M.D. Anderson Cancer Center UTHealth Graduate School of Biomedical Sciences, Houston, Texas.

^3^ Current address: Baylor College of Medicine, Houston, Texas.

^*^Corresponding author: anne.marie.krachler@uth.tmc.edu

**Contains:**

Figures S1-S7

**Supplemental Movies**

SI movie 1: AB movie.AVI

SI movie 2: SOX10 movie.AVI

SI movie 3: Atropine movie.AVI


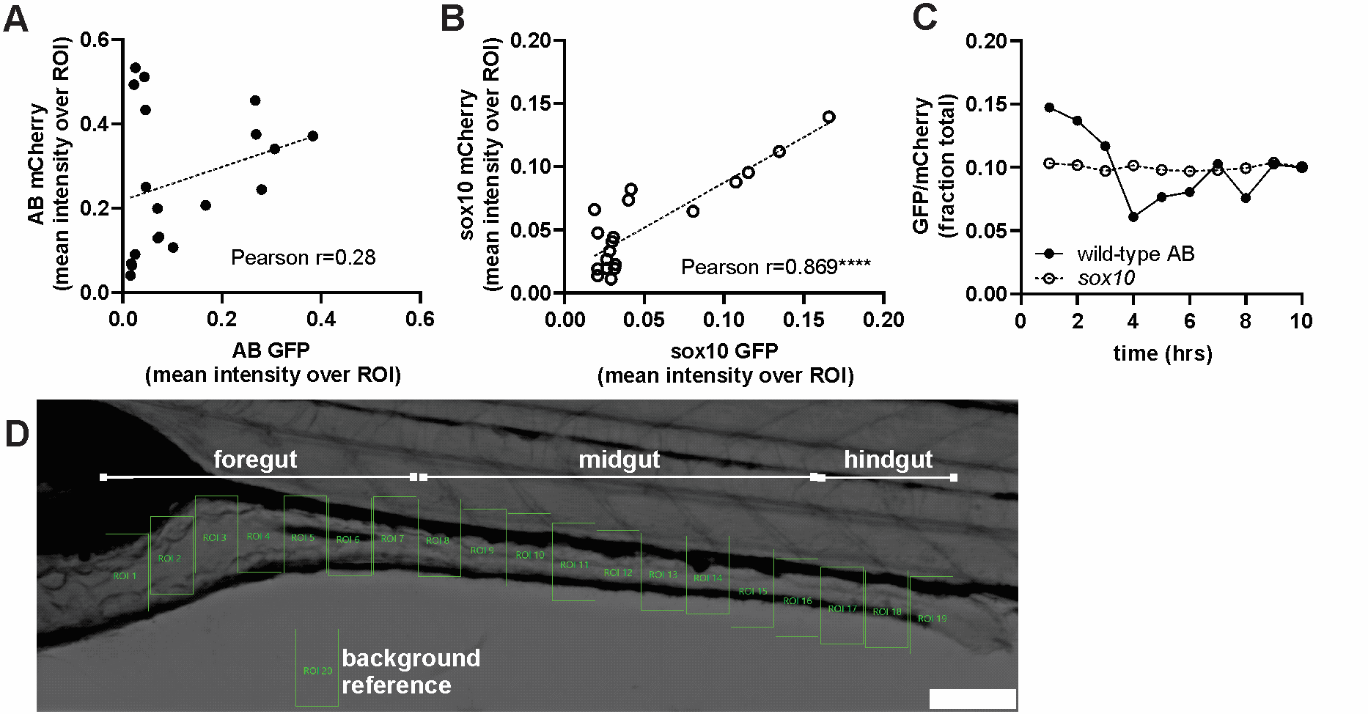


**Figure S1. Correlation and temporal dynamics of *ler:gfp* expression relative to bacterial density.** Mean GFP intensities were plotted against mean mCherry intensities across all larvae and time points, for each ROI (1-19), for wild-type **(A)** and *sox10* fish **(B)**, and linear correlation (dashed lines), and Pearson correlation coefficients were determined. **(C)** GFP/mCherry intensity ratios for wild-type AB (solid black) and *sox10* (empty circles, dashed line) fish were averaged across larvae and ROIs 1-19 to analyze fluctuations over time. **(D)** Example of ROI placement for intensity quantification in respect to larval intestine. Foregut (ROI 1-7), midgut (ROI 8-16), hindgut (ROI 17-19), and reference for background correction (ROI 20). Scale bar 100 µm.


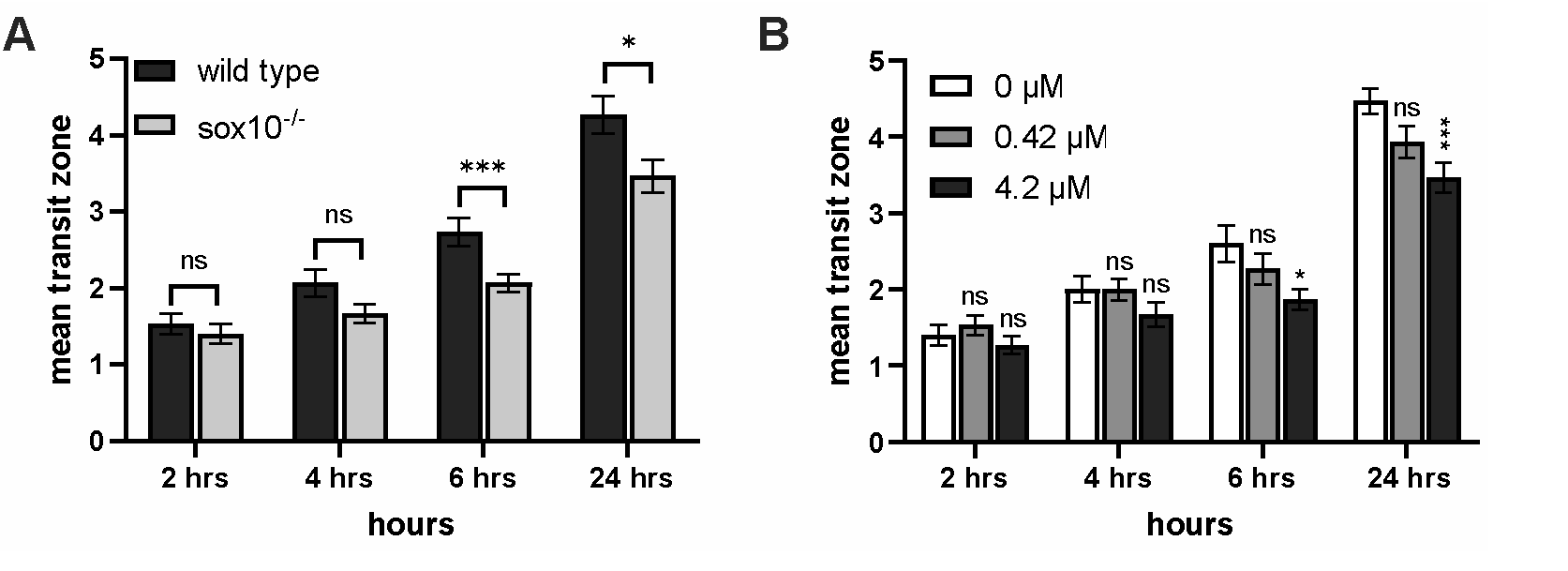


**Figure S2. Gut motility is impaired in *sox10* larvae and following atropine treatment. 6 dpf wild-type and *sox10* larvae (A) or 6 dpf wild-type larvae treated with 0-4.2 µM atropine during the experiment (B) were fed fluorescent tracer food. Fish with tracer-filled bulb were sorted into batches of n=5/group (3 independent experiments, total n=15 fish/group) and fish were scored at 2, 4, 6, and 24 hours to determine the most anterior transit zone still occupied with fluorescent food. Data are mean transit zone scores ± sem, and wild-type vs *sox10* mutant at each time point (A) were compared by unpaired t-tests, treated vs untreated groups (B) at each time point were compared by ANOVA. 0.42 µM atropine does not significantly alter transit;** ***p≤0.001, *p<0.05, ns (not significant) p≥0.05;


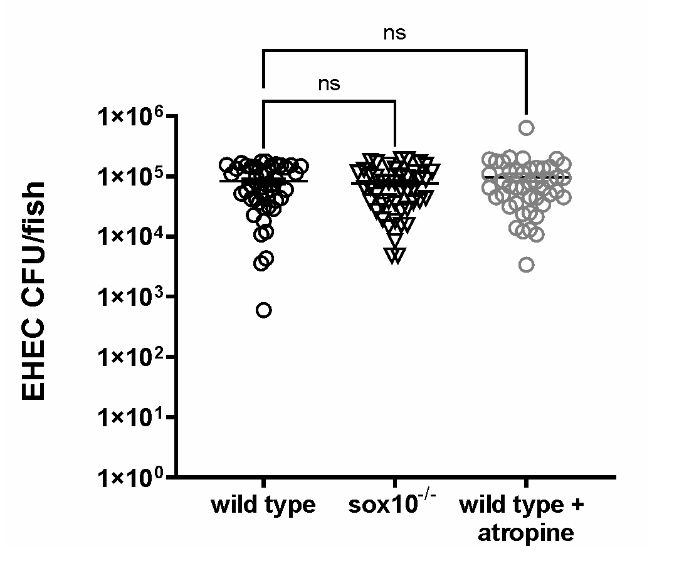


**Figure S3. EHEC initial burden/uptake in wild-type vs *sox10* zebrafish line vs wild-type fish treated with atropine.** Results are EHEC CFUs recovered from individual fish (n=45 fish/group over 3 independent experiments) immediately following the 2 hrs paramecia incubation, and means ± sem. Groups were compared to wild-type using one-way ANOVA and Dunnett’s test for multiple comparisons. Ns, no significant differences (p≥0.05).


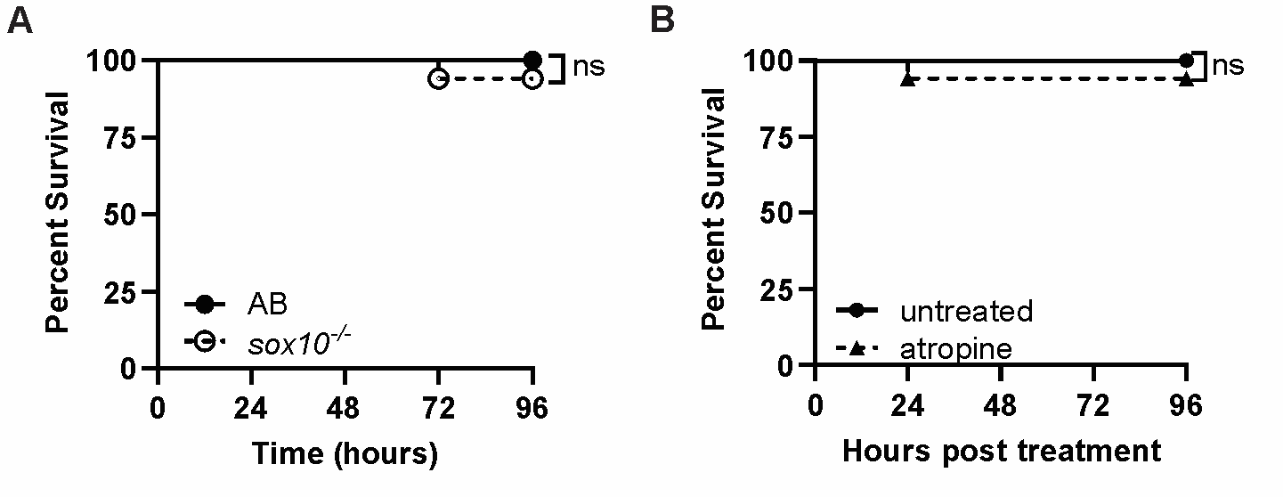


**Figure S4. Survival of uninfected larvae.** Survival of uninfected **(A)** wild-type AB versus *sox10* larvae and **(B)** untreated AB vs AB treated with 4.2 µM atropine (n=144 fish/group over 3 independent experiments) was analyzed using Kaplan-Meier plots. *Sox10* and atropine-treated groups, respectively, were compared to wild-type AB using a Mantel-Cox test. ns, no significant difference between groups (p≥0.05);


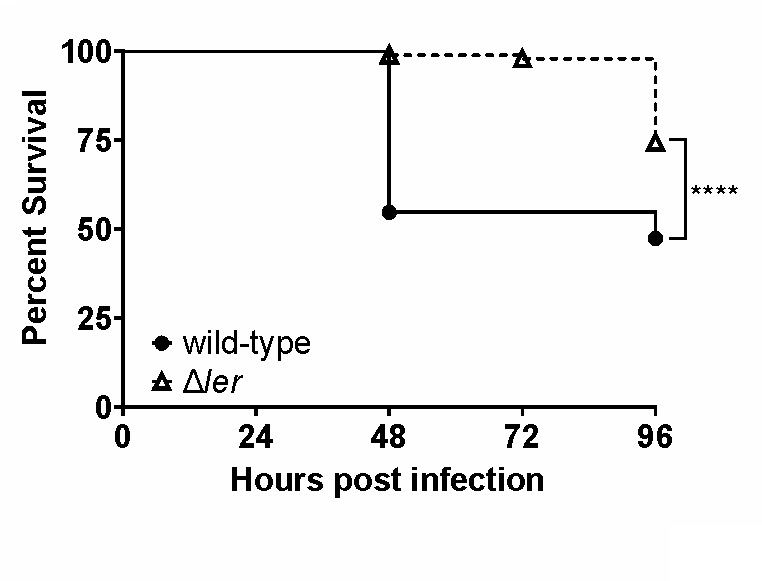


**Figure S5. Disruption of T3SS activity attenuates infection *in vivo*.** Survival od wild-type larvae infected with high dose of wild-type EHEC or Δ*ler* mutant (n≥18 fish/group over 3 independent experiments) was analyzed using Kaplan-Meier plots. Results were compared using a log-rank (Mantel-Cox) test. ****p≤0.0001;


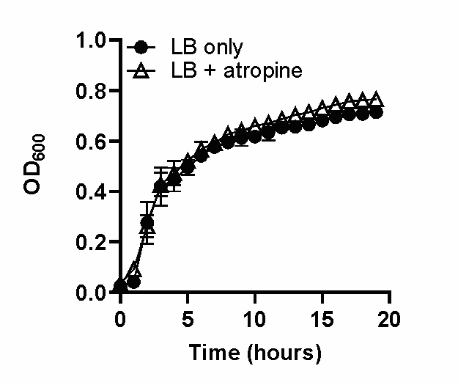


**Figure S6. Atropine treatment does not significantly affect EHEC growth.** Growth of EHEC strain 86-24 in LB with or without 4.2 µM atropine was determined by measuring OD600 every hour for 19 hours using a FluoStarOmega plate reader. Throughout the experiment, cultures were incubated at 37 °C at 120 rpm. Data are means ± sem from at least three independent experiments.


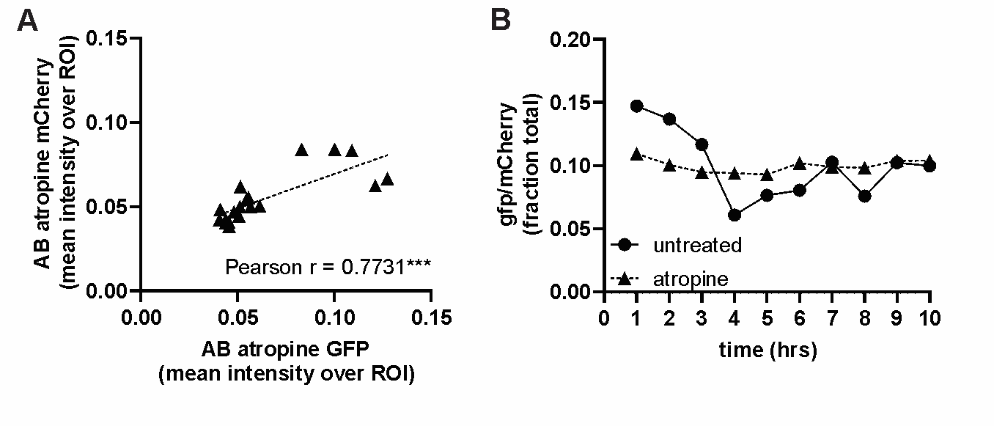

**Figure S7. Correlation and temporal analysis of *ler:gfp* expression relative to bacterial density in atropine-treated larvae. (A)** Mean GFP intensities were plotted against mean mCherry intensities across all larvae and time points, for each ROI (1-19). Linear correlation (dashed line) and Pearson’s r value are shown. **(B)** GFP/mCherry intensity ratios for untreated (circles) and atropine-treated (triangles, dashed line) wild-type AB were averaged across larvae and ROIs 1-19 to visualize temporal fluctuation of fluorescence intensity.
